# Detection of high serum levels of β-D-Glucan in disseminated nocardial infection: a case report

**DOI:** 10.1186/s12879-017-2370-4

**Published:** 2017-04-13

**Authors:** Toyomitsu Sawai, Takumi Nakao, Shota Yamaguchi, Sumako Yoshioka, Nobuko Matsuo, Naofumi Suyama, Katsunori Yanagihara, Hiroshi Mukae

**Affiliations:** 1 Department of Respiratory Medicine, Nagasaki Harbor Medical Center City Hospital, 6-39 Shinchi-machi, Nagasaki, 850-8555 Japan; 2 Department of Laboratory Medicine, Nagasaki Harbor Medical Center City Hospital, 6-39 Shinchi-machi, Nagasaki, Japan; 3 Department of Laboratory Medicine, Nagasaki University Hospital, 1-7-1 Sakamoto-machi, Nagasaki, Japan; 4 Second Department of Internal Medicine, Nagasaki University Hospital, 1-7-1 Sakamoto-machi, Nagasaki, Japan

**Keywords:** Nocardial infection, β-D-glucan, Cross-reactivity, Serum

## Abstract

**Background:**

β-D-glucan (BDG) is a helpful diagnostic marker for many invasive fungal infections, but not for nocardiosis. Here, we reported the first case of nocardial infection with high serum level of BDG.

**Case presentation:**

A 73-year-old man was hospitalized because of fever, headache, and appetite loss after 10 months of steroid and immunosuppressive therapy for cryptogenic organizing pneumonia. With a diagnosis of bacterial pneumonia, treatment with ampicillin/sulbactam was initiated. There was improvement on chest radiograph, but fever persisted. Further work-up revealed multiple brain abscesses on cranial magnetic resonance imaging (MRI). Serum galactomannan and BDG were elevated at 0.6 index and 94.7 pg/ml, respectively. Voriconazole was initiated for presumed aspergillus brain abscess. However, fever persisted and consciousness level deteriorated. Drainage of brain abscess was performed; based on the Gram stain and Kinyoun acid-fast stain, disseminated nocardiosis was diagnosed. Voriconazole was then shifter to trimethoprim/sulfamethoxazole. The presence of *Nocardia farcinica* was confirmed by the 16S rRNA gene sequence. Treatment course was continued; BDG level normalized after 1 month and cranial MRI showed almost complete improvement after 2 months.

**Conclusion:**

BDG assay is widely used to diagnose invasive fungal infection; therefore, clinicians should be aware that *Nocardia* species may show cross-reactivity with BDG assay on serum.

## Background

*Nocardia* species is a gram-positive, branching, filamentous bacillus that may cause localized and disseminated infections in humans, including pulmonary infection, subcutaneous infection, brain abscess, and bacteremia. β-D-glucan (BDG) is a polysaccharide glucose polymer that is found in a broad range of fungal agents, but not in the cell wall of *Nocardia* species. Therefore, BDG is not thought to be useful in diagnosing nocardiosis. However, Koncan et al. reported a case of *Nocardia abscessus* brain abscess that showed elevated level of BDG on cerebrospinal fluid [1]. They also described that *Nocardia* species showed cross-reactivity with BDG assay. Here, we presented the first reported case of nocardial infection with elevated serum BDG level.

## Case presentation

**Fig. 1:**
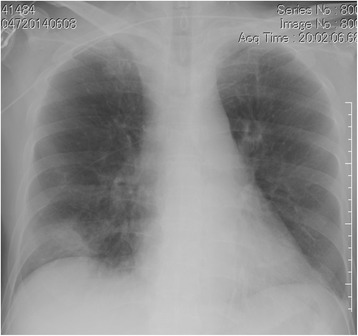
Chest radiography on admission showed infiltration shadow in the right lower lung field

**Fig. 2:**
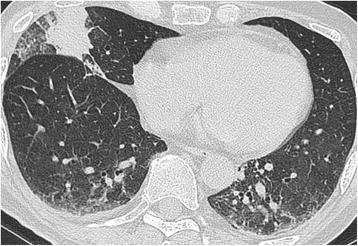
Chest CT on admission showed infiltration shadow in the right middle lobe and ground glass opacity in both lower lobes

**Fig. 3:**
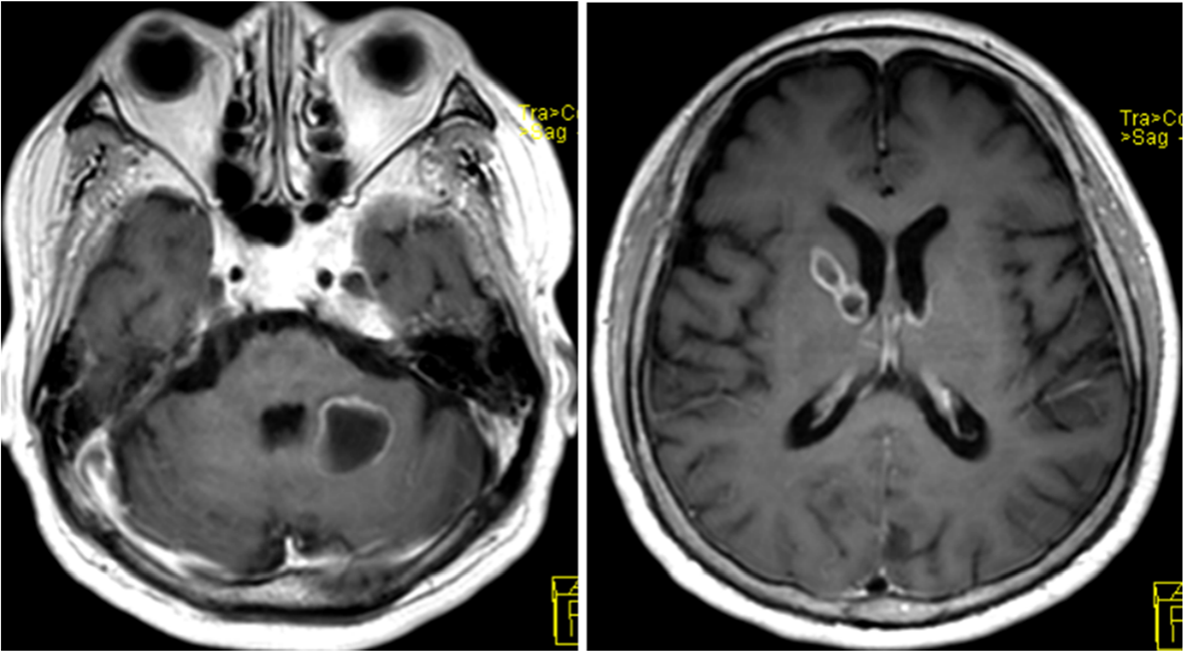
Cranial MRI showed multiple masses

**Fig. 4:**
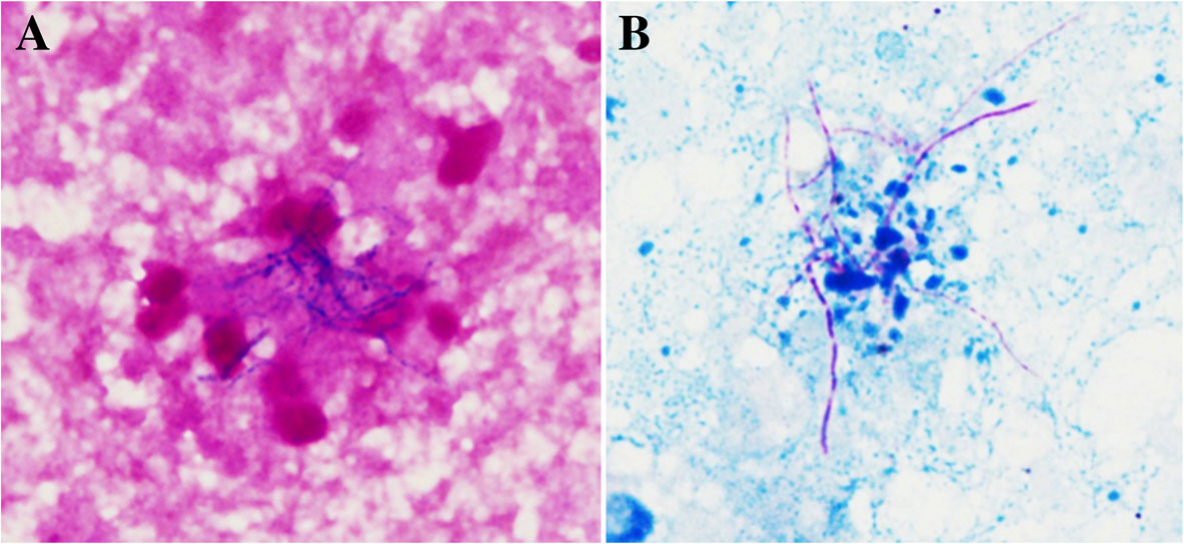
**a** Gram stain of abscess (×1000) showed a cluster of branching, filamentous Gram-positive rods. **b** Modified acid fast Kinyoun staining of abscess (×1000) showed filamentous red-stained partially acid-fast rods

A 73-year-old man, Asian, and ex-smoker presented to our department with complaints of fever, headache and appetite loss after 10 months of steroid and immunosuppressive therapy for cryptogenic organizing pneumonia. Physical examination revealed a heart rate of 88 beats/min, blood pressure of 142/74 mmHg, respiratory rate of 20 breaths/min, temperature of 38.5 °C, and oxygen saturation of 95% on room air. Chest examination revealed coarse crackles on right lower lung field. Chest radiograph and computed tomography showed infiltrates on the right middle lobe (Figs. 1 and 2). The white blood cell count, C-reactive protein, and procalcitonin levels were 15,100 /μl, 5.73 mg/dl, and 0.221 ng/ml, respectively. Test for human immunodeficiency virus (HIV) infection was negative. Expectorated sputum smears were negative for bacteria and acid-fast bacilli. Urinary antigen test (Binax NOW; Binax, Inc., Portland, ME) for *Streptococcus pneumoniae* and *Legionella pneumophila* were negative. Although it was difficult to determine the causative bacteria, a presumptive diagnosis of community-acquired pneumonia was made, and treatment with ampicillin/sulbactam (ABPC/SBT) 1.5 g intravenously every 6 h was initiated. On day 8, white blood cell count normalized and chest radiograph showed improvement; however, fever persisted. Due to a suspicion of drug fever, ABPC/SBT was discontinued. However, fever persisted even after 4 days of ABPC/SBT discontinuation. To search for the source of infection, cranial magnetic resonance imaging (MRI) was done and showed multiple masses (Fig. 3); brain abscess was diagnosed. Serum Quantiferon test (QuantiFERON-TB Gold In-Tube; Cellestis, Chadstone, Australia), cryptococcus antigen and toxoplasma antibody were negative. Galactomannan (Platelia Aspergillus Ag; Bio-Rad, Marnes-la-Coquette, France) and β-D-glucan (Wako; Wako Pure Chemical Industries, Ltd., Tokyo, Japan) were elevated at 0.6 index, and 94.7 pg/ml, respectively. On day 11, voriconazole (VRCZ) 200 mg intravenously every 12 h was initiated for presumed aspergillus brain abscess. However, after 5 days of treatment, fever continued and consciousness level deteriorated. On day 16, drainage of brain abscess was performed; Gram stain revealed many neutrophils and gram-positive rods with branching, filamentous hyphae (Fig. 4a). Kinyoun acid-fast stain of the gram-positive rods was positive (Fig. 4b). On the basis of these findings, disseminated nocardiosis was diagnosed, and VRCZ was shifted to intravenous trimethoprim/sulfamethoxazole (TMP/SMX) 240 mg/1200 mg every 8 h. The cultured strain was sent to Department of Laboratory medicine of Nagasaki University Hospital, where the presence of *Nocardia farcinica* was confirmed by 16S rRNA gene sequence. Treatment course was continued. BDG level normalized after 1 month, therefore intravenous TMP/SMX was shifted to oral TMP/SMX 240 mg/1200 mg every 8 h. Cranial MRI showed almost complete improvement after 2 months. Unfortunately, the patient died from aspiration pneumonia after 6 months of treatment.

## Discussion

*Nocardia* infections are rare, but potentially fatal conditions that are typically more problematic in patients with cell-mediated immunosuppression [2], but may occasionally affect immunocompetent patients as well [3]. *Nocardia* species is a ubiquitous, gram-positive, actinomycete saprophyte that lives in soil, organic matter and water. Human infection usually arises from direct inoculation of the skin or by inhalation. It is considered an opportunistic pathogen that affects patients with impaired cell-mediated immunity. Most of the patients with *Nocardia* infection have predisposing factors, such as hematologic malignancies, treatment with corticosteroids, solid tumors, bone marrow or solid organ transplantation, HIV infection or chronic pulmonary or renal disease [2]. The most common clinical presentation is subacute pneumonia with nodules, necrosis, cavitary disease, lung abscess, effusion, and/or empyema [4–6]. Nocardial brain abscess is a rare clinical finding, most of which originate from a primary focus, such as the lungs, skin, or elsewhere in the body [7]. Central nervous system involvement was recognized in over 44% of all cases of systemic nocardiosis [8]. Moreover, nocardial brain abscess carries the highest mortality rate among all bacterial cerebral abscesses [9].

Due to the nonspecific clinical and radiologic manifestations, including irregular nodules, cavitation, reticulonodular shadow, infiltration and pleural effusion, pulmonary nocardiosis is easily misdiagnosed as bacterial pneumonia, tuberculosis, mycosis or actinomycosis [10]. Furthermore, nocardial brain abscess is often mistaken for aspergillosis, tuberculosis, or malignancy because of the similar clinical manifestations [11]. Therefore, helpful diagnostic markers, such as Quantiferon test, cryptococcus antigen, galactomannan, and β-D-glucan (BDG) are very useful. BDG is a polysaccharide glucose polymer that is found in a broad range of commonly encountered fungal agents, including *Candida* spp., *Aspergillus* spp., and *Pneumocystis jirovecii*, with notable exception of cryptococci, zygomycetes, and *Blastomyces dermatitidis*. BDG is helpful for the diagnosis of invasive fungal infections. This test is a chromogenic, quantitative enzyme immunoassay designed to detect BDG using purified, lysed amebocytes (Limulus amebocyte lysate). These cells contain components of the Limulus clotting cascade, including factors C and G, which initiate coagulation in the presence of bacterial lipopolysaccharide and BDG, respectively. By removing factor C from the lysate, the manufacturers limit activation of the cascade to BDG alone. However, false-positive results with certain hemodialysis filters [12], fungal derived antibiotics [13], immunoglobulins [14], *Alcaligenes faecalis* and *Stretococcus pneumoniae* infection [15], and bacteremia caused by *Pseudomonas aeruginosa* [16] have been described. Koncan et al. reported a case of *Nocardia abscessus* brain abscess with BDG level that was elevated in the cerebrospinal fluid, but negative in serum [1]. To our best knowledge, nocardial infection with high serum level of BDG has not been reported previously. Our case was the first English article about nocardial infection with elevated BDG level on serum. All known causes of false-positive BDG assay, including invasive fungal disease, hemodialysis treatment using cellulose membrane filter, and use of fungal-derived antibiotics and immunoglobulins, were not present in this patient. Metan et al. reported that clinical administration of intravenous ABPC/SBT caused false-positive BDG assay, although not statistically significant [17]. On the other hand, Marty et al. reported that ABPC/SBT tested negative for BDG at the drug-infusate concentration or at maximum plasma concentration [18]. In our patient, BDG level was examined 4 days after ABPC/SBT discontinuation; we think that ABPC/SBT administration did not cause a false-positive BDG assay. In this patient, because the bacterial suspension of the clinical isolate of *Nocardia farcinica* was positive for BDG, environmental contamination was unlikely the cause of the positive serum BDG assay. We tested with BDG assay both a strain of *N. farcinica* isolated from brain pus sample in this case and pure blood agr as negative control used Wako assay (Wako; Wako Pure Chemical Industries, Ltd., Tokyo, Japan). The positive cut off was 11 pg/ml. The strain was cultured on blood agr plate for 24 h at 37 °C. The bacteria were suspended in glucan free sterile saline and harvested by centrifugation (3000×g, 4 °C, 5 min). The organisms were resuspended in glucan free sterile saline and diluted from 1 × 10^8^ to 2 × 10^8^ CFU/ml as estimated by turbidimetry. Pure blood agar as negative control were always negative, but BDG levels of *N. farcinica* were mildly elevated to about 20 pg/ml.

Pulmonary nocardiosis and nocardial brain abscess can be easily misdiagnosed clinically. Moreover, culture of *Nocardia* species is difficult because of the slow growth of the bacteria and the presence of normal flora in the culture. Microbiologic diagnosis of nocardiosis and identification of nocardia clinical isolates by conventional methods are difficult and time-consuming [19]. Additionally, cross-reactivity of *Nocardia* species with serum BDG assay makes the diagnosis of nocardiosis difficult. Because BDG assay is widely used to diagnose invasive fungal infection, clinicians should take into consideration the fact that *Nocardia* species may show cross-reactivity with BDG assay on serum. Clinicians should aim to detect the causative microbes without excessively depending on helpful serologic tests, such as BDG.

## Conclusion

In conclusion, the first case of nocardial infection with high serum level of BDG was described. Clinicians should be aware of the possibility that *Nocardia* species may show cross-reactivity with BDG on serum.

## Abbreviations

ABPC/SBT: Ampicillin/sulbactam
BDG: β-D-glucan
TMP/SMX: Trimethoprim/sulfamethoxazole
VRCZ: Voriconazole
